# Antimicrobial, antioxidant, and cytotoxic properties of endophytic fungi isolated from *Thysanolaena maxima* Roxb., *Dracaena spicata* Roxb. and *Aglaonema hookerianum* Schott.

**DOI:** 10.1186/s12906-023-04185-4

**Published:** 2023-09-30

**Authors:** Nazia Hoque, Zihan Rahman Khan, Parisa Tamannur Rashid, Mst. Nadira Begum, Suriya Sharmin, Md. Jamal Hossain, Md. Sohel Rana, Md. Hossain Sohrab

**Affiliations:** 1 Department of Pharmacy, East West University, Aftabnagar, Dhaka, 1212 Bangladesh; 2 Pharmaceutical Sciences Research Division, Bangladesh Council of Scientific and Industrial Research (BCSIR), Dhaka, 1205 Bangladesh; 3 Department of Pharmacy, Jahangirnagar University, Savar, Dhaka, 1342 Bangladesh; 4 Biological Research Division, Bangladesh Council of Scientific and Industrial Research (BCSIR), Dhaka, 1205 Bangladesh; 5 Department of Pharmacy, State University of Bangladesh, 77 Satmasjid Road, Dhanmondi, Dhaka, 1205 Bangladesh

**Keywords:** Endophytic fungi, Morphology, Antimicrobial, Antioxidant, Cytotoxic properties

## Abstract

**Background:**

Endophytic fungi have recently been recognized as an impressive source of natural biomolecules. The primary objective of the research was to isolate fungal endophytes from *Thysanolaena maxima* Roxb., *Dracaena spicata* Roxb. and *Aglaonema hookerianum* Schott. of Bangladesh and assess their pharmacological potentialities focusing on antimicrobial, antioxidant, and cytotoxic properties.

**Methods:**

The fungal isolates were identified up to the genus level by analyzing their macroscopic and microscopic characteristics. Ethyl acetate extracts of all the fungal isolates were screened for different bioactivities, including antimicrobial (disc diffusion method), antioxidant (DPPH scavenging assay), and cytotoxic (brine shrimp lethality bioassay) activities.

**Results:**

Among the thirteen isolates, *Fusarium* sp. was the most recognized genus, while the others belonged to *Colletotrichum* sp. and *Pestalotia* sp. Comparing the bioactivity of all the extracts, *Fusarium* sp. was shown to be the most effective endophyte, followed by *Colletotrichum* sp. and *Pestalotia* sp. In the antimicrobial study, two isolates of *Fusarium* sp. (internal strain nos. DSLE-1 and AHPE-4) showed inhibitory activity against all the tested bacteria and the highest zone of inhibition (15.5 ± 0.4 mm) was exerted by AHPE-4 against *Bacillus subtillis*. All the fungal isolates produced mild to moderate free radical scavenging activity, where the highest antioxidant activity was revealed by one isolate of *Fusarium* sp. (internal strain no. AHPE-3) with an IC_50_ value of 84.94 ± 0.41 µg/mL. The majority of *Fusarium* sp. isolates exhibited notable cytotoxic activity, where AHPE-4 exhibited the highest cytotoxicity, having the LC_50_ value of 14.33 ± 4.5 µg/mL.

**Conclusion:**

The findings of the study endorsed that the fungal endophytes isolated from *T. maxima*, *D. spicata*, and *A. hookerianum* hold potential as valuable origins of bioactive substances. Nevertheless, more comprehensive research is warranted, which could develop novel natural compounds from these endophytes to treat various infectious and cancerous diseases.

## Background

Endophytes are often asymptomatic microorganisms, including bacteria or fungi, that inhabit the internal plant tissues [1]. This coevolutionary process of the endophytic fungi and its symbiotic plants creates excellent attraction to the researcher for their prime involvement in novel drug discovery. Fungal endophytes have tremendous biosynthetic capability, allowing them to synthesize bioactive secondary metabolites with unique characteristics [2]. They are mostly reported to contain valuable bioactive compounds such as quinones, coumarins, isocoumarins, alkaloids, anthraquinones, naphthoquinones, terpenoids, steroids, lignans, and lactones [3, 4]. These bioactive substances have been reported to display a variety of biological activities, including antiparasitic, antimicrobial, antiviral, anti-inflammatory, anticancer, antioxidant and immunosuppressive activities [5, 6]. Endophytic fungi possess diverse taxonomic groups prevalently distributed within plants, playing numerous roles in plant health and productivity [7, 8]. The fungal taxonomy is mainly based on morphological characteristics, from which primary recognition of species or genera can be predicted.

Chittagong Hill Tracts (CHT) are large hilly areas in Bangladesh consisting of rich forest composition. The indigenous domestic people in this hilly region are invariably relying on wild medicinal plants to resolve their therapeutic purposes [9]. *Thysanolaena maxima* Roxb., *Dracaena spicata* Roxb., and *Aglaonema hookerianum* Schott. are renowned medicinal mountainous plants of CHT, Bangladesh. *T. maxima* has been used in treating of eye infections, tonsillitis, boils, and skin diseases by the tribal population of Bangladesh and India [10–12]. Traditional healers of different tribes of CHT use leaf juice of *D. spicata* in the treatment of fever, cold, coughs, and measles [13, 14]. *A. hookerianum* has been used for the treatment of hemorrhoids, arthritis, gout, conjunctivitis, constipation, and hysteria [15]. These ethnopharmacologically important plants and their secondary metabolites are also reported to have potential pharmacological properties, including antioxidant, antimicrobial, cytotoxic, analgesic, and CNS depressant activities [10–12, 15].

Endophytes can produce bioactive metabolites similar to their host plants and are capable of showing similar bioactivity [16]. As the aforementioned plants have intriguing ethnomedicinal properties, it is anticipated that the associated endophytic fungi may exhibit potential bioactivity in addition to their ability to produce a wide range of bioactive chemicals. Therefore, it is necessary to identify and investigate the potential endophytic fungal diversity of the selected medicinal plants. Moreover, the identification of prospective fungi using morphological analysis provides an opportunity to look for further analysis regarding novel compound investigation [17]. The present study describes the morphological characterization and bioactivity of endophytic fungi isolated from three well-known ethnomedicinal plants in hilly areas of Bangladesh.

## Methods

### Materials

DPPH (2,2-Diphenyl-1-picrylhydrazyl) was purchased from Sigma-Aldrich Co., USA. Potato dextrose agar media, water agar media, nutrient agar media, standard disc of kanamycin and ketoconazole were purchased from Hi media, India. All the chemicals and solvents used were of analytical grades.

### Collection of plant samples

Fresh plant samples of *T. maxima, D. spicata*, and *A. hookerianum* were collected from Rangamati, CHT, Bangladesh, with proper permission from the local authority. Plant samples of *T. maxima*, *D. spicata* and *A. hookerianum* were identified by a taxonomist, Sarder Nasir Uddin, Principal Scientific Officer, Bangladesh National Herbarium, Dhaka, Bangladesh, with accession nos.: DACB 42267, DACB 40632 and DACB 40633, respectively. The voucher specimens of the plants have been deposited in the herbarium for further reference. The research methodology involving the plant materials was approved by the Research Committee of the Department of Pharmacy, Jahangirnagar University, Savar, Dhaka.

### Isolation and extraction of endophytes

Endophytic fungi were isolated from fresh and healthy plant tissues (flower, stem, leaf, bark and petiole) of *T. maxima*, *D. spicata* and *A. hookerianum* using the surface sterilization method [18]. The respective plant parts were washed, cut into smaller pieces and surface-sterilized by immersing the plant parts into 70% ethanol, 1.3 M sodium hypochlorite, and 70% ethanol sequentially. The surface-sterilized plant parts were dried and placed on separate Petri dishes containing water agar (WA) medium. Streptomycin (100 mg/L) was mixed with the WA medium to inhibit the growth of endophytic bacteria. For the control study, unsterilized plant segments were also prepared simultaneously to isolate the surface-contaminating fungi. Petri dishes were incubated at 28 ± 2 °C for fungal growth for 4–6 weeks. The fungal hyphae grown on the plant segments after the incubation period were isolated and transferred onto potato dextrose agar (PDA) medium for subculture.

A total of 5 endophytic fungi were isolated from the plant *T. maxima* of which TMFE-1, TMFE-2 and TMFE-3 were isolated from flower stems and TMLE-2 and TMLE-3 were isolated from the leaves of the plant. Similarly, 4 endophytic fungi were isolated from *D. spicata* of which DSLE-1, DSLE-2 and DSLE-4 were isolated from leaves and DSBE-1 was isolated from the bark of the plant. On the other hand, 4 endophytic fungi were isolated from *A. hookerianum* of which AHLE-1 and AHLE-4 were isolated from leaves and AHPE-3 and AHPE-4 were isolated from petioles of the plant. All the isolated fungal endophytes were cultivated on PDA medium for 21 days at room temperature. The culture medium for each fungus was then extracted with ethyl acetate for 7 days to obtain the crude extracts. After filtration and solvent evaporation, the extracts yielded a crude mixture of microbial secondary metabolites [19].

### Morphological identification of endophytes

Isolated endophytes were identified on the basis of morphological features following macroscopic and microscopic characteristics using standard identification manuals [20]. For macroscopic identification, the specific morphology of the fungal colonies (e.g., color, mycelia, hyphae, margin, texture, growth rate etc.) was observed. For microscopic identification, the Lactophenol Cotton Blue (LPCB) staining method was followed to prepare the slides from the cultures to observe the spore characteristics [21].

### Antimicrobial activity

The extracts of endophytic fungi were tested for antimicrobial activity following the disc diffusion method [22]. All the fungal extracts were tested against four gram-positive bacteria including *Bacillus cereus* (ATCC 14579), *Bacillus megaterium* (ATCC 25918), *Bacillus subtilis* (ATCC 6059), and *Staphylococcus aureus* (ATCC 25923) and six gram-negative bacteria including *Salmonella typhi* (ATCC 13311), *Escherichia coli* (ATCC 28739), *Vibrio mimicus* (ATCC 33653), *Shigella dysenteriae* (ATCC 26131), *Shigella boydii* (ATCC 13147) and *Pseudomonas aeruginosa* (ATCC 27833). To determine the antifungal activity, two pathogenic fungi *Aspergillus flavus* and *Aspergillus niger* were used. The test microorganisms were inoculated on nutrient agar (for bacteria) and PDA medium (for fungi). The test samples (crude fungal extracts) were prepared (100 µg/disc) and kanamycin (30 µg/disc) and ketoconazole (30 µg/disc) standard discs were used as the reference. The inoculated strains were incubated at 37 ± 2 °C (for bacteria) and 25 ± 2 °C (for fungi) for 24 h for their optimum growth. The antimicrobial activity of the samples was determined by measuring the diameter of the zone of inhibition produced by the samples in millimeters (mm).

### Antioxidant activity

The DPPH free radical scavenging activity of all the fungal extracts was tested to determine the antioxidant activity [23]. To measure the effectiveness, varying concentrations of test samples dissolved in methanol were prepared from 200.0 µg/mL to 12.5 µg/mL using the serial dilution method. For the control study, ascorbic acid and methanol were used following similar sample preparations. The absorbance of the samples was measured at 517 nm using methanol as a blank. Inhibition of free radical DPPH in percent (%) was calculated as follows:

$$\%\, of\, scavenging = (1 - A_{sample} / A_{control}) \times 100$$

where A_control_ is the absorbance of the control which contains all reagents excluding the samples. The concentration at which the sample provides 50% inhibition (IC_50_) indicating the scavenging activity, was calculated from the graph plotted against the concentration of the extracts.

### Cytotoxic activity

All fungal extracts were examined for preliminary cytotoxicity following the brine shrimp lethality bioassay [24]. The brine shrimp eggs were allowed to hatch for 24 h in seawater to be matured as nauplii. Test tubes were prepared to contain 5 mL of seawater along with different sample solutions prepared from 400 µg/mL to 0.39062 µg/mL using dimethyl sulfoxide (DMSO) and 10 living nauplii were added to each of the test tubes. For the control group, samples of vincristine sulfate and DMSO were prepared in the same manner. After 24 h, the number of surviving nauplii was counted and the 50% lethal concentration (LC_50_) of each sample was calculated by linear correlation obtained from the graph of the logarithm of concentration against the percentage of mortality.

## Results

### Morphological identification of isolated fungi

**Fig. 1:**
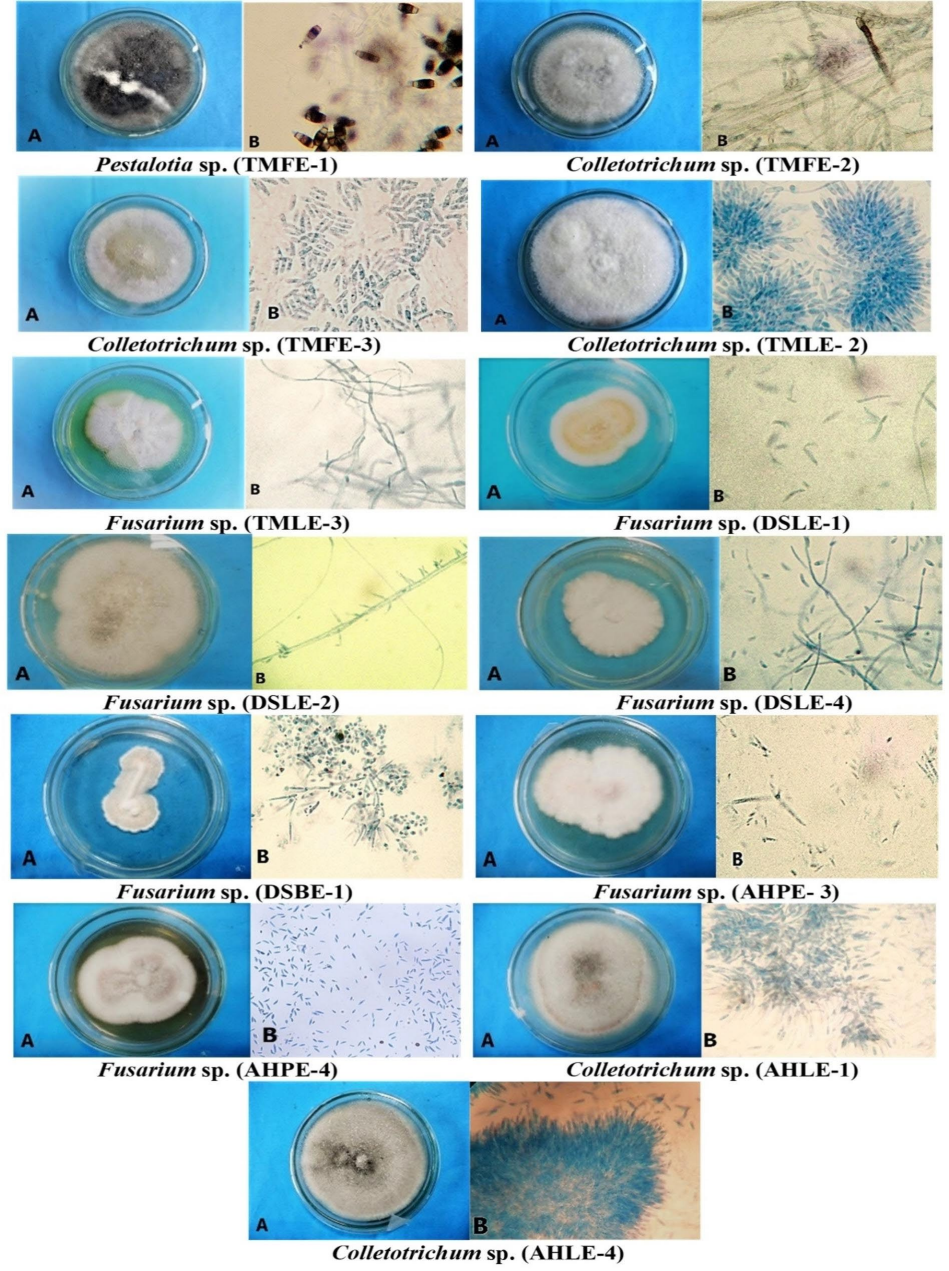
Macroscopic and microscopic characteristics of isolated endophytic fungi; (**A**) Macroscopic view (**B**) Microscopic view (40x)

A total of thirteen endophytic fungi were isolated from *T. maxima*, *D. spicata* and *A. hookerianum* (Fig. 1). Among the isolates, seven isolates belonged to *Fusarium* sp. (TMLE-3, DSLE-1, DSLE-2, DSLE-4, DSBE-1, AHPE-3 and AHPE-4), five isolates belonged to *Colletotrichum* sp. (TMFE-2, TMFE-3, TMLE-2, AHLE-1 and AHLE-4) and only one isolate was identified as *Pestalotia* sp. (TMFE-1). Identification of the genus was based on macroscopic and microscopic characteristics broadly described in Tables 1 and 2 and confirmed according to the previous morphological investigation of the genus [25–27].

**Table 1:** Macroscopic characteristics of isolated endophytes

Identified genus	Internal strain no.	Colony Morphology					
		Margin	Texture	Growth rate	Hyphae	Mycelia	Color of the reverse side
*Pestalotia* sp.	TMFE-1	Entire	Radial	Fast	Surficial	Cottony and aerial	Black
*Colletotrichum* sp.	TMFE-2	Filiform	Radial	Fast	Aerial	Cottony, filamentous and raised at the center	White
*Colletotrichum* sp.	TMFE-3	Entire	Radial	Fast	Aerial	Velvety and slightly flat at the center	White
*Colletotrichum* sp.	TMLE-2	Filiform	Wooly	Moderate	Aerial	Raised and fibrous	Off white
*Fusarium* sp.	TMLE-3	Undulate	Radial	Slow	Aerial	Soft and elevated at the center	Purple
*Fusarium* sp.	DSLE-1	Undulate	Radial	Moderate	Surficial	Soft and grows upward	Light pink
*Fusarium* sp.	DSLE-2	Lobate	Wooly	Moderate to rapid	Aerial	Cottony and dense near center	Off white
*Fusarium* sp.	DSLE-4	Entire	Wooly	Slow	Surficial	Smooth and slightly raised	Off white
*Fusarium* sp.	DSBE-1	Lobate	Wooly	Slow	Aerial	Elevated and velvety	Off white
*Fusarium* sp.	AHPE-3	Undulate	Wooly	Moderate	Surficial	Densely floccose at the center	Pinkish white
*Fusarium* sp.	AHPE-4	Undulate	Radial	Moderate	Surficial	Elevated and fluffy	Pink
*Colletotrichum* sp.	AHLE-1	Lobate	Wooly	Fast	Aerial	Cottony, dense and aerial mycelium	Gray
*Colletotrichum* sp.	AHLE-4	Entire	Wooly	Fast	Aerial	Soft, dense and aerial mycelium	Black and white border

**Table 2:** Microscopic characteristics of the isolated endophytes

Identified genus	Internal strain no.	Microscopically visible features		
		Mycelium	Conidia	Conidiophores
*Pestalotia* sp.	TMFE-1	Branched appendages	Dark and relatively broad conidia. Straight, ellipsoid and septate	Short and simple
*Colletotrichum* sp.	TMFE-2	Septate and hair-like structures	Cylindrical, aseptate Presence of seta	Short, simple, hyaline
*Colletotrichum* sp.	TMFE-3	Septate, highly branched	Single-celled cylindrical attenuated with a rounded end. Presence of seta	Short and simple
*Colletotrichum* sp.	TMLE-2	Highly branched	Large, single-celled conidia with abundant sporulation rate	Short, simple and dense
*Fusarium* sp.	TMLE-3	Mass branched	Curved and septate	Short and simple
*Fusarium* sp.	DSLE-1	Branched	Macroconidia 3–5 celled	Short, simple and hyaline
*Fusarium* sp.	DSLE-2	Branched and hyaline	Single-celled microconidia	Short and simple
*Fusarium* sp.	DSLE-4	Branched and hyaline	Long sporodochial Conidia, slightly curved	Irregularly branched
*Fusarium* sp.	DSBE-1	Branched	Small microconidia formed from phialides	Short, irregularly branched
*Fusarium* sp.	AHPE-3	Branched	Single-celled microconidia. Macroconidia Fusiform, septate	Monophialidic
*Fusarium* sp.	AHPE-4	Branched	Single-celled microconidia Oblong and septate	Short, simple and hyaline
*Colletotrichum* sp.	AHLE-1	Finely branched	Falciform. Long, single-celled conidia	Short and simple
*Colletotrichum* sp.	AHLE-4	Septate	Small, single-celled conidia	Septate, hyaline

### Antimicrobial activity

In the antimicrobial screening, the isolated fungal extracts showed mild to moderate activities (8–15.5 mm) against all the tested bacteria and fungi (Table 3). Among the isolates, DSLE-1 (*Fusarium* sp.) and AHPE-4 (*Fusarium* sp.) showed inhibitory activity against all the gram-positive and gram-negative bacteria while TMLE-2 (*Colletotrichum* sp.), TMLE-3 (*Fusarium* sp.), DSLE-1 (*Fusarium* sp.), DSBE-1 (*Fusarium* sp.) and AHPE-4 (*Fusarium* sp.) showed inhibitory activity against the gram-positive bacteria only. The extract of AHPE-4 (*Fusarium* sp.) showed the highest antibacterial activity (15.5 ± 0.4 mm) against *Bacillus subtilis* compared to the standard kanamycin. In determining antifungal activity, DSBE-1 (*Fusarium* sp.), AHPE-3 (*Fusarium* sp.) and AHPE-4 (*Fusarium* sp.) showed inhibition against *Aspergillus niger* where DSBE-1 showed the highest antifungal activity (13.3 ± 0.2 mm). The zone of inhibition < 7 mm produced by the fungal extracts was considered inactive against microorganisms.

**Table 3:** Antimicrobial activity (zone of inhibition) of the isolated endophytic fungi

Microorganisms	*Pestalotia* sp. (TMFE-1)	*Colletotrichum* sp. (TMFE-2)	*Colletotrichum* sp. (TMFE-3)	*Colletotrichum* sp. (TMLE-2)	*Fusarium* sp. (TMLE-3)	*Fusarium* sp. (DSLE-1)	*Fusarium* sp. (DSLE-2)	*Fusarium* sp. (DSLE-4)	*Fusarium* sp. (DSBE-1)	*Fusarium* sp. (AHPE-3)	*Fusarium* sp. (AHPE-4)	*Colletotrichum* sp. (AHLE-1)	*Colletotrichum* sp. (AHLE-4)	Kanamycin	Ketoconazole
	100 µg/disc													30 µg/disc	
**Gram-positive bacteria**															
*Bacillus megaterium*	10.3 ± 0.4			10.6 ± 0.4	8.8 ± 0.6	10.6 ± 0.4			10.3 ± 0.4	9.5 ± 0.4	13.5 ± 0.4			30.6 ± 0.4	ND
*Bacillus subtilis*				11.3 ± 0.4	10.6 ± 0.2	9.6 ± 0.4			12.5 ± 0.4		15.5 ± 0.4			34.5 ± 0.4	ND
*Staphylococcus aureus*	11 ± 0.8			9.6 ± 0.4	10.6 ± 0.4	13.5 ± 0.4			10.6 ± 0.2		14.6 ± 0.4			32 ± 0.0	ND
*Bacillus cereus*	9.6 ± 0.4			11.6 ± 0.4	13.5 ± 0.4	10.8 ± 0.6			8.8 ± 0.6		12.8 ± 0.4			31.5 ± 0.4	ND
**Gram-negative bacteria**															
*Escherichia coli*	8.8 ± 0.6			10.6 ± 0.4	12.8 ± 0.6	12.8 ± 0.6			13.5 ± 0.4	9 ± 0.0	14.3 ± 0.4		13.5 ± 0.4	33.8 ± 0.2	ND
*Pseudomonas aeruginosa*	9.5 ± 0.4			7.6 ± 0.4	11 ± 0.8	11.6 ± 0.4			12.8 ± 0.6	9.3 ± 0.2	12.8 ± 0.6			35.3 ± 0.4	ND
*Salmonella typhi*				12.5 ± 0.4		9.6 ± 0.4					15.1 ± 0.2			35 ± 0.4	ND
*Vibrio mimicus*				10.8 ± 0.6		11.3 ± 0.4					12.0 ± 0.0			31.6 ± 0.4	ND
*Shigella boydii*						11 ± 0.8					12.1 ± 0.2			17.3 ± 0.4	ND
*Shigella dysenteriae*						10.6 ± 0.4					11.3 ± 0.4			19.5 ± 0.4	ND
**Fungi**															
*Aspergillus flavus*														ND	43.3 ± 0.4
*Aspergillus niger*									13.3 ± 0.2	9 ± 0.0	10.8 ± 0.6			ND	41 ± 0.8

Values are expressed as mean ± SD; n = 3; ‘-’ means no activity; ND = Not done; Codes in the parentheses represent internal strain nos. of the fungal endophytes.

### Antioxidant activity

All the fungal extracts showed free radical scavenging activity in our present study (Table 4). However, AHPE-3 (*Fusarium* sp.) exhibited the lowest IC_50_ value of 84.94 ± 0.41 µg/mL indicating slightly potent antioxidant activity compared to the standard ascorbic acid (2.38 ± 0.75 µg/mL).

**Table 4:** Antioxidant activity of the isolated endophytic fungi

Name of the samples	IC_50_ values (µg/mL)	Name of the samples	IC_50_ values (µg/mL)
*Pestalotia* sp. (TMFE-1)	192.6 ± 0.3	*Fusarium* sp. (DSLE-4)	112 ± 0.98
*Colletotrichum* sp. (TMFE-2)	188.28 ± 1.98	*Fusarium* sp. (DSBE-1)	124.17 ± 0.03
*Colletotrichum* sp. (TMFE-3)	190.26 ± 1.05	*Fusarium* sp. (AHPE-3)	84.94 ± 0.41
*Colletotrichum* sp. (TMLE-2)	147.1 ± 0.28	*Fusarium* sp. (AHPE-4)	107.74 ± 0.22
*Fusarium* sp. (TMLE-3)	124.3 ± 0.63	*Colletotrichum* sp. (AHLE-1)	147.05 ± 0.52
*Fusarium* sp. (DSLE-1)	132.5 ± 0.57	*Colletotrichum* sp. (AHLE-4)	137.07 ± 1.48
*Fusarium* sp. (DSLE-2)	130.19 ± 0.13	Ascorbic acid	2.38 ± 0.75

Values are expressed as mean ± SD; n = 3; Codes in the parentheses represent internal strain nos. of the fungal endophytes

### Cytotoxic activity

**Fig. 2:**
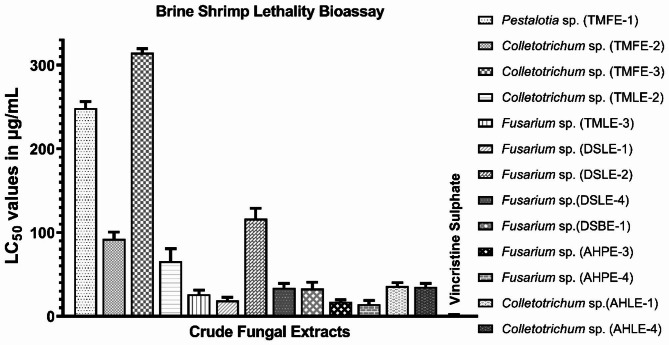
Cytotoxic activity of the isolated endophytic fungi. Values are expressed as mean ± SD; n = 3; Codes in the parentheses represent internal strain nos. of the fungal endophytes

In the brine shrimp lethality bioassay, TMLE-3 (*Fusarium* sp.), DSLE-1 (*Fusarium* sp.), AHPE-3 (*Fusarium* sp.) and AHPE-4 (*Fusarium* sp.) showed potent cytotoxicity with LC_50_ values of 25.98 ± 5.2 µg/mL, 18.88 ± 3.84 µg/mL, 17.15 ± 2.4 µg/mL and 14.33 ± 4.5 µg/mL, respectively compared to the standard vincristine sulfate (1.63 ± 0.44 µg/mL). The rest of the fungal extracts showed mild to moderate cytotoxic activity (Fig. 2).

### Chemical screening

Chemical screening of all the fungal isolates was conducted by thin-layer chromatography (TLC) to evaluate the presence of various secondary metabolites. The screening of all the extracts was executed by visual observation, under UV light (254 and 365 nm) and after spraying with vanillin-H_2_SO_4_ spray reagent (Table 5). Analysis of the TLC spots of the extracts showed the presence of diversified secondary metabolites such as coumarins, isocoumarins, or their derivatives, flavonoids, steroids, terpenoids, anthocyanins, anthraquinones and naphthoquinones [28, 29].

**Table 5:** Chemical screening of fungal extracts by thin-layer chromatography

Identified genus	Internal strain no.	Visual observation	Visibility under UV light (254 nm)	Visibility under UV light (365 nm)	Visibility with spray reagent	Prospective compounds
*Pestalotia* sp.	TMFE-1		Blue quenching Dark quenching	Blue Sky blue Greenish yellow	Dark purple	Steroids, Terpenoids Coumarin, Isocoumarin or their derivatives
*Colletotrichum* sp.	TMFE-2		Blue quenching Dark quenching	Greenish yellow Blue Sky blue	Dark purple Bluish purple	Steroids, Terpenoids Coumarin, Isocoumarin or their derivatives
*Colletotrichum* sp.	TMFE-3		Dark quenching	Greenish yellow Light yellow	Pink Dark purple	Flavonoids or their derivatives, Terpenoids
*Colletotrichum* sp.	TMLE-2		Blue quenching Dark quenching	Violet Sky blue	Purple Bluish purple	Coumarin, Isocoumarin or their derivatives Terpenoids, Steroids
*Fusarium* sp.	TMLE-3		Light quenching Dark quenching	Blue Red Yellow Sky blue Violet	Dark purple	Coumarins Anthocyanins Terpenoids Steroids
*Fusarium* sp.	DSLE-1		Blue Sky blue Violet Dark quenching	Yellow Violet Blue	Dark purple	Coumarin, Isocoumarin or their derivatives Flavonoids or their derivatives, Terpenoids Steroids
*Fusarium* sp.	DSLE-2		Dark quenching Light quenching	Blue	Dark purple Magenta	Terpenoids, Steroids Coumarin, Isocoumarin or their derivatives
*Fusarium* sp.	DSLE-4		Dark quenching	Violet Blue	Pink	Terpenoids Steroids Anthraquinones Coumarin, Isocoumarin or their derivatives
*Fusarium* sp.	DSBE-1		Dark quenching Dark blue Light sky blue	Light yellow Blue Sky blue	Dark purple	Coumarin, Isocoumarin or their derivatives Terpenoids Steroids
*Fusarium* sp.	AHPE-3		Light sky blue Light blue quenching	Sky blue Dark blue	Dark purple	Coumarin, Isocoumarin or their derivatives, Terpenoids, Steroids
*Fusarium* sp.	AHPE-4	Pink Reddish purple	Light blue Dark quenching	Sky blue Dark blue	Dark purple	Coumarin, Isocoumarin or their derivatives Naphthoquinones, Anthraquinone derivatives Terpenoids, Steroids
*Colletotrichum* sp.	AHLE-1		Dark quenching Sky blue	Light green Sky blue Blue	Pink Purple	Terpenoids Steroids Coumarin, Isocoumarin or their derivatives
*Colletotrichum* sp.	AHLE-4		Dark quenching Sky blue	Light green, Sky blue, Blue	Dark purple	Terpenoids, Steroids Coumarin, Isocoumarin or their derivatives

## Discussion

This study aimed to determine the presence and explore the pharmacological activities of the endophytic fungi isolated from three different medicinal plants, *T*. *maxima*, *D*. *spicata*, and *A*. *hookerianum*, which are widely distributed in the hill tracts of Bangladesh. These plants are an integral part of folklore medicine with sufficient scientific proof of persistent pharmacological activities [30]. Our study led to the isolation of thirteen taxonomically recognized fungal endophytes belonging to *Fusarium* sp., *Colletotrichum* sp., and *Pestalotia* sp. The fungal isolates were characterized morphologically based on the data obtained from macroscopic and microscopic analyses and comparing those features with authentic identification manuals [20, 31].

All the crude fungal extracts were analyzed for their in vitro antimicrobial, antioxidant, and cytotoxic activities. Most of the extracts of *Fusarium* sp. displayed inhibition against both gram-positive and gram-negative bacteria and pathogenic fungi. One isolate of *Fusarium* sp. (AHPE-4) showed notable antibacterial activity against *B. subtilis* (15.5 ± 0.4 mm), *S. typhi* (15.1 ± 0.2 mm), *S. aureus* (14.6 ± 0.4 mm) and *E. coli* (14.3 ± 0.4 mm). Another isolate of *Fusarium* sp. (DSBE-1) produced antifungal activity against *A. niger* producing a zone of inhibition of 13.3 ± 0.2 mm. This trait supports the potential of the compounds of *Fusarium* sp. for the development of antimicrobial agents against several pathogenic bacteria and fungi. *Fusarium* sp. is one of the most potential fungal genera and has the ability to produce diversified bioactive secondary metabolites due to having many unique gene clusters [32]. Chemical screening of the extracts of *Fusarium* sp. also confirms the presence of coumarins, terpenoids, and quinones, which are reported to have antimicrobial activities [33]. Some previous studies [34, 35] reported that promising antimicrobial compounds such as fusaric acid, bikaverin, dehydrofusaric acid and beauvericin were isolated from different endophytic genera of *Fusarium* sp. Fusaric acid, a recognized mycotoxin, potentially displays antimicrobial effects by directly regulating the transcription of several genes associated with the pyocyanin pathway in *Pseudomonas* sp. On the other hand, bikaverin functions as an antimicrobial agent by hindering the DNA and protein synthesis processes within microorganisms. Hence, the existence of these phytochemicals might be accountable for the antimicrobial effects of *Fusarium* sp. However, further comprehensive investigations are necessary to achieve a complete understanding of this phenomenon.

In the present study, the crude extract of *Fusarium* sp. (AHPE-3) exhibited moderate DPPH free radical scavenging activity with IC_50_ values of 84.94 ± 0.41 µg/mL. Several previous studies also reported the antioxidant effects of endophytic *Fusarium* sp. [36, 37]. Natural antioxidants such as polyphenolic chemicals and flavonoids contain multiple hydroxyl groups which allow them to transfer an electron to unstable free radical DPPH and reduce oxidative stress [38]. Numerous coumarins were shown to have antioxidant activities by influencing the generation and scavenging of reactive oxygen species [39]. The presence of coumarins, flavonoids and/or their derivatives in the extracts might be responsible for exerting such antioxidant activity [40]. However, the rest of the fungal extracts exhibited mild radical scavenging activity in this study. Future studies should be conducted to establish the antioxidant potential of the fungal endophytes through other methods which could establish more specific antioxidative pathways for the specific biomolecules.

To evaluate the preliminary cytotoxicity of the samples, brine shrimp lethality bioassay has been serving as a popular tool as it is simple, affordable, and requires no specialized equipment or aseptic environment [41]. In the present study, most of the extracts of *Fusarium* sp. (AHPE-3, AHPE-4, DSBE-1, DSLE-4, and TMLE-3) demonstrated potent cytotoxicity producing LC_50_ values from 14.33 ± 4.5 µg/mL to 25.98 ± 5.2 µg/mL. Literature studies have shown that *Fusarium* sp. can produce numerous mycotoxins such as enniatins, fusaric acid, fumonisin, and moniliformin with strong cytotoxic activities [42]. Moreover, the presence of coumarins, isocoumarins, terpenoids, naphthoquinone, and anthraquinone was established by the TLC analysis of the extracts and these compounds were also reported to have potential cytotoxicity. However, a more specific investigation is required to establish the relation between the cytotoxicity and the reported secondary metabolites.

Our study has presented the endophytic fungal species of three ethnomedicinal plants of Bangladesh as the alternate ecological resources of bioactive molecules. To the best of our knowledge, this is the first study on the endophytic fungal flora associated with these plants in Bangladesh, which opens an unexplored area for further research.

## Conclusion

This investigation presents remarkable data about the morphology and pharmacological activities of the fungal endophytes isolated from the mentioned three medicinal plants of Bangladesh. The findings of the study established *Fusarium* sp. as one of the prospective endophytes because of their significant cytotoxicity and antimicrobial activity, in addition to moderate antioxidant activity. TLC analysis revealed the existence of diverse secondary metabolites in all the crude fungal extracts. Further comprehensive research on the specific endophytes is needed to discover new bioactive compounds that could be effective in combating infections and cancers.

## Data Availability

Further raw data involved in this study are available on request from the corresponding authors.
